# Effects of Endocrine Interventions Targeting ERα or PR on Breast Cancer Risk in the General Population and Carriers of *BRCA1/2* Pathogenic Variants

**DOI:** 10.3390/ijms25115894

**Published:** 2024-05-28

**Authors:** Deborah Huber, Maria Hatzipanagiotou, Susanne Schüler-Toprak, Olaf Ortmann, Oliver Treeck

**Affiliations:** 1 Department of Gynecology and Obstetrics, University Medical Center Regensburg, 93053 Regensburg, Germany; deborahrahel.huber@mri.tum.de (D.H.); mhatzipanagiotou@csj.de (M.H.); sschueler@csj.de (S.S.-T.); olaf.ortmann@klinik.uni-regensburg.de (O.O.); 2 Department of Obstetrics and Gynecology, Technical University of Munich, 80333 Munich, Germany

**Keywords:** *BRCA1/2* mutation, hormonal contraception, hormone replacement therapy, breast cancer risk, estrogen metabolism

## Abstract

There is evidence suggesting that endocrine interventions such as hormone replacement therapy and hormonal contraception can increase breast cancer (BC) risk. Sexual steroid hormones like estrogens have long been known for their adverse effects on BC development and progression via binding to estrogen receptor (ER) α. Thus, in recent years, endocrine interventions that include estrogens have been discussed more and more critically, and their impact on different BC subgroups has increasingly gained interest. Carriers of pathogenic variants in *BRCA1/2* genes are known to have a high risk of developing BC and ovarian cancer. However, there remain open questions to what extent endocrine interventions targeting ERα or the progesterone receptor further increase cancer risk in this subgroup. This review article aims to provide an overview and update on the effects of endocrine interventions on breast cancer risk in the general population in comparison to *BRCA1/2* mutation carriers. Finally, future directions of research are addressed, to further improve the understanding of the effects of endocrine interventions on high-risk pathogenic variant carriers.

## 1. Introduction

Endocrine interventions such as hormone replacement therapy (HRT) and hormonal contraception (HC) have become increasingly common over the years. Several studies suggested that HRT use can increase the risk for breast cancer (BC) [1,2] and ovarian cancer (OC) [3]. Regarding HC use, studies have suggested a potential increase in BC risk and a decrease in OC risk [2,4]. Thus, the risks and benefits of such interventions have to be considered carefully by the administering physicians. This is particularly relevant for women who already face a higher cancer risk, especially carriers of *BRCA1/2* pathogenic variants. However, regarding the effect of endocrine interventions on this high-risk BC subgroup, there remain a lot of uncertainties, both for patients and attending physicians [5]. In this subgroup, the cumulative risk of developing breast cancer up to the age of 80 years is about 72% in *BRCA1* and 69% in BRCA2 pathogenic variant carriers [6]. Ovarian cancer risks are 44 and 17%, respectively [7]. As ovarian cancer is still the most lethal gynecological cancer and is mainly diagnosed in its advanced stages, timely risk-reducing bilateral salpingoophorectomy (RRBSO) is recommended for *BRCA1/2* mutation carriers. Risk-reducing surgery is generally recommended at the age of 40 or up to 5 years before the earliest recorded age of onset of ovarian cancer in the family, bringing forward the need to consider hormone substitution. Consequently, possible adverse effects on breast cancer risk from HRT have to be weighed against the negative effects of estrogen loss in premenopausal women.

In this review article, we aim to provide an overview and update on the impact of HRT and HC on breast cancer risk in the general population compared to their effects on carriers of *BRCA1/2* pathogenic variants. In vitro fertilization (IVF) is also an important endocrine intervention that might affect cancer risk; however, since data on IVF, particularly regarding cancer risk for *BRCA1/2* mutation carriers, are sparse, we decided to focus on HRT and HC. For this purpose, we searched the PubMed database from 1995 to December 2023 using the terms “hormonal contraception“, “oral contraception“, and “hormone replacement therapy“ in combination with “breast cancer“ or “BRCA mutation“. Amongst the research articles with new insights within the described topic, the most relevant studies with a possible impact on clinical practice were selected.

## 2. Hormones and Breast Cancer

### 2.1. Endogenous Hormones and Breast Cancer

BC is the most frequently diagnosed cancer in females, with an estimated 2.3 million incident annual cases worldwide as of 2020. Furthermore, it represents the leading cause of cancer mortality in women [8]. HR-positive BC cases account for approximately 80% [9]. Regarding BC risk factors, several studies have shown a modifying influence of exogenous as well as endogenous hormones.

Not only the development, but also post-puberal regulation of the breast tissue is affected by different hormones. Besides the most important female sex steroid hormones being estrogens, progesterone, and prolactin, the physiology of the mammary gland is also determined by other hormones like insulin, thyroxine, and cortisol, as well as different growth factors [10].

Estrogens play a major role in growth promotion of both the mammary gland and breast cancer tissue, particularly by activating estrogen receptor α (ERα), which is known to induce expression of cell cycle promoters and anti-apoptotic genes. This receptor acts as an estrogen-inducible transcription factor binding to estrogen response elements (EREs) in the regulatory promoter regions of a variety of estrogen-responsive genes, thereby activating gene expression of, e.g., genes coding for the cell cycle regulators Cyclin D1 and Cyclin A2, and activating the expression of anti-apoptotic protein BCL2. Since ligand-bound ERα is also able to induce expression of progesterone receptor (PR) in normal and malignant breast tissue, usually ERα and PR are co-expressed [11]. However, in comparison to the mammary gland, ERα is overexpressed in ERα-positive BC, thereby promoting tumor growth. Accordingly, higher levels of ERα in healthy breast epithelium seem to be associated with an increased risk of breast cancer development [12]. Since ERα plays a major tumor-promoting role, it has been an important target in breast cancer treatment for many years [13].

Given the anti-estrogenic effect of progesterone, it was proposed several decades ago that breast cancer risk might be associated with a defective luteal phase, resulting in lower progesterone levels and a predominance of estrogen [14]. Accordingly, it has been observed that increased serum levels of free estradiol in postmenopausal women are associated with a substantially elevated breast cancer risk, up to a relative risk (RR) of 2.58 [15]. Regarding other estrogens and androgens (including estrone, androstenedione, dehydroepiandrosterone, and testosterone), a similar extent of increased breast cancer risk has been observed, while an inverse association has been shown for sex hormone-binding globulin (SHBG) [15]. A later analysis within the European Prospective Investigation into Cancer and Nutrition (EPIC) cohort showed a positive association of breast cancer cases expressing ERα, classically referred to as hormone-receptor (HR)-positive breast cancer, with higher estrogen and androgen serum levels in postmenopausal women [16]. The association was stronger for HR-positive than for HR-negative breast cancer: the odds ratio (OR) for the highest versus the lowest tertile of estradiol was 2.91 (95% CI, 1.62–5.23; *p* trend = 0.002) for HR-positive and 2.11 (95% CI, 1.00–4.46; *p* trend = 0.05) for HR-negative breast cancer, respectively. The association of higher levels of androgens and breast cancer risk might be explained by the aromatization of testosterone and androstenedione into estrone and estradiol. The local conversion of androgens into estrogens within breast cancer tissue may lead to higher local estradiol levels, possibly further promoting cell proliferation, especially in HR-positive breast cancer cells [16].

As estrogen levels are higher in obese women, due to the effect of the enzyme aromatase in the adipose tissue, and this observation coincides with the increase in breast cancer risk for postmenopausal women with higher body mass index (BMI) [17]. In addition, the California Teachers Study, a prospective cohort study published in 2012, examined the association between BMI and HR-positive and -negative breast cancer [18]. The study found an increase in risk for HR-positive, but not for HR-negative, breast cancer associated with postmenopausal obesity.

The association between estrogens, androgens, and breast cancer risk in premenopausal women was examined in 2006 by a large case-control study nested within the Nurses’ Health Study II [19]. Similarly to postmenopausal women, higher levels of estradiol were also associated with an increase in breast cancer risk up to an RR of 2.4 [19]. The association was stronger for HR-positive breast cancers, but only remained statistically significant when tested for all invasive breast cancers. Regarding androgens, higher levels of testosterone and androstenedione were also associated with an increase in invasive breast cancer risk. Again, the association was more pronounced for HR-positive breast cancer.

Estrogen signaling in the mammary gland and in BC is not only mediated by binding of estrogens to ERα, but also to the second nuclear estrogen receptor, ERβ, and the membrane G-protein coupled estrogen receptor GPER1 [20]. ERβ is the dominant ER in the mammary gland and is expressed in more than 50% of BC cases, neither depending on ERα status nor the molecular BC subtype, and is considered a tumor suppressor and partial ERα antagonist in BC and prostate cancer [21]. In the case of co-expression, ERα/ERβ ratio is considered to notably affect growth of breast epithelial and BC cells. The role of GPER1 in BC remains controversial, being reported to exert both tumor-promoting and tumor-suppressing functions, but survival analyses of large datasets including GeneChip RNA microarray data of 4929 BC patients supported GPER1 acting as a tumor suppressor in this cancer entity, with a significantly longer RFS for patients with high GPER1 expression [22].

While the proliferative effect of estradiol on epithelial cells of the mammary gland has been widely accepted, the impact of endogenous progesterone on breast cancer risk has been controversial throughout the last few years [23]. As mentioned above, progesterone has formerly been perceived as an antiestrogen, thus with a primarily anti-proliferative function [14]. However, it has been shown that the highest proliferation within mammary epithelial cells during the menstrual cycle in premenopausal women is observed during the luteal phase, which is characterized by higher progesterone than estrogen levels [14]. Former experimental data suggest both pro- and anti-carcinogenic effects of progesterone metabolites, suggesting breast cancer risk might be influenced by the balance of these factors [24]. Later, the identification of PR splice variants PR-A and PR-B with differential effects on breast cancer progression increased the complexity of PR signaling [25]. In luminal BC expressing ERα and therefore high levels of PR, PR-A triggered invasiveness and metastasis and was shown to be associated with lymph node involvement. In contrast, PR-B only increased metastasis in the presence of high progesterone concentrations, and no association with lymph node involvement was observed [26]. The ATAC trial showed that breast cancer patients with a higher ratio of PR-A to PR-B expression experienced earlier relapse under treatment with tamoxifen [27]. However, separating the effects of estrogens and progesterone on the mammary gland is complicated, due to the strong interactions and codependency between estrogen and progesterone signaling. Indeed, since the expression of the progesterone receptor (PR) in the gland tissue is activated by ERα, the interaction of the two hormones may be more important than the distinction of separate effects [11].

A recent cohort study found an increase in breast cancer risk by 16% for postmenopausal women with higher circulating progesterone levels (hazard ratio (HR) = 1.16; 95% CI, 1.00–1.35; *p* = 0.048) [23]. The association was not modified by other factors such as age or BMI. However, the extent of the breast cancer risk modification associated with progesterone was dependent on circulating estradiol levels: women with increased serum progesterone levels had an increased breast cancer risk when estradiol levels were higher, but a reduced breast cancer risk when the circulating estradiol levels were very low. These findings emphasize the dependency of progesterone and estrogen regarding their effect on the mammary gland: higher progesterone levels might possibly enhance the proliferative effect induced by higher serum estradiol. The authors of the study further concluded that relatively higher progesterone levels compared to estradiol levels could have a weak antimitotic effect, resulting in the observed reduction in breast cancer risk when estradiol levels were very low. This, however, interferes with the highest proliferation of epithelial breast tissue cells taking place during the luteal phase, as mentioned above. One possible explanation might be a different impact of sex hormones on the mammary gland in pre- compared to postmenopausal women. On the other hand, previous experimental data have shown that the transcription of PR in the mammary is largely dependent on ERα expression [28]. Thus, in cases of very low estradiol levels, progesterone might not be able to unfold its proliferative effect, due to a lower expression of PR.

### 2.2. Hormone Replacement Therapy (HRT) and Breast Cancer

More than 50% of all postmenopausal women experience climacteric symptoms such as hot flashes [29]. Despite HRT being the most effective treatment, indications and possibly harmful side effects have been observed more critically since the publication of the Women’s Health Initiative (WHI) study in 2002. Besides risk factors for cardiovascular adverse events, a possible increase in cancer risks has to be taken into consideration. Regarding breast cancer, the different impacts of estrogen-only (ET) and combined estrogen-progestin therapy (EPT) have to be distinguished.

Several studies have described an increase in breast cancer risk associated with EPT administration in postmenopausal women. The WHI study remains the world’s largest randomized-controlled trial (RCT) to date. The trial showed an increase in breast cancer risk (HR = 1.25; 95% CI, 1.07–1.46; *p* = 0.004) after a mean duration of use of 5.6 years associated with continuous application of conjugated equine estrogens (CEE) and medroxyprogesterone acetate (MPA) [30]. Furthermore, an increase in breast cancer mortality was observed (HR = 1.96; 95% CI, 1.00–4.04; *p* = 0.049). Breast cancer risk was slightly higher when HRT was started less than five years after menopause (*p* = 0.08). There was no significant difference in histology or hormone receptor expression between the intervention and the placebo group. In fact, triple-negative tumors or overexpression of HER2 were observed more often in the intervention group, although these differences were not statistically significant. However, breast cancers in the intervention group were more often node-positive (*p* = 0.03), consistent with the unfavorable outcome regarding breast cancer specific mortality. In a cumulative follow-up after 18 years, however, the increase in breast cancer mortality did not remain statistically significant (HR = 1.44, 95% CI, 0.97–2.15; *p* = 0.07) [1].

Regarding ET, the WHI trial did not find an increased breast cancer risk associated with CEE-only therapy (HR = 0.75; 95% CI, 0.51–1.09), whereby the numbers were considerably lower compared to the EPT analysis [31]. In the 18-year follow-up, breast cancer mortality was reduced in the CEE group (HR = 0.55; 95% CI, 0.33–0.92; *p* = 0.02) [1]. In a further re-analysis of data from the two randomized studies of the WHI, a significantly lower risk of breast cancer (HR 0.78; 95% CI 0.65–0.93; *p* = 0.005) and a significantly lower risk of breast cancer mortality (HR 0.60; 95% CI 0.37–0.97; *p* = 0.04) were found in the ET arm [32]. In contrast, EPT led to a significantly higher risk of breast cancer (HR 1.28; 95% CI 1.13–1.45; *p* < 0.001), but had no effect on breast-cancer-specific mortality (HR 1.35; 95% CI 0.94–1.95; *p* = 0.11) [32].

A more recent meta-analysis of the available prospective data conducted by the Collaborative Group on Hormonal Factors in Breast Cancer showed an increase in breast cancer risk associated with all types of HRT, except vaginal ET [33]. Risk increased with the duration of use and did not remain elevated past use when HRT had not been taken longer than 4 years. However, for current users, a significant increase was already observed after less than five years. Increases in risk were consistently higher for EPT than for ET, with the highest RR observed for current users during years 5–14, being 2.08 (2.02–2.15) and 1.33 (1.28–1.37), respectively. No relevant difference between the different age groups was observed. However, for women who started HRT at 60–69 years, the association with breast cancer risk remained significant only for EPT, but not for ET.

Regarding the different regimen of HRT, EPT with continuous application of progestagen was associated with the highest increase in risk. However, no substantial difference was observed for different progestagenic constituents. Regarding the containing estrogen, risks did not significantly differ for different types or applications of estrogens.

Increase in risk associated with current use during years 5–14 was significantly higher for ER-positive and lobular tumors than for ER-negative and ductal tumor types.

Concerning obesity as an additional risk factor, the Collaborative Group on Hormonal Factors in Breast Cancer found a positive association between BMI and breast cancer risk in never users, but not in current users of HRT [33]. ER-positive breast cancer was mainly responsible, not only for the association between HRT and breast cancer risk in current users, but also for the association between BMI and breast cancer risk in never users. As mentioned above, postmenopausal obesity led to an increase in HR-positive breast cancer risk [18]. In obese women, the addition of exogenic hormones did not seem to further increase relative breast cancer risks. This can be explained by the increased endogenous estrogenic stimulation of breast tissue in obese women, caused by aromatization of androgens in the fatty tissue.

The reason for the higher impact of EPT on breast cancer risk when compared to ET is not yet completely understood. Due to the known tumor-promoting actions of estrogens, the exogenous supply of this hormone would be expected to be mainly responsible for the increase in breast cancer risk associated with HRT. However, the current data clearly suggest that the progestagen component has a considerable influence on breast cancer risk. Given that expression of the nuclear PR variants A and B is induced by estrogens, which have been shown to exert distinct effects on breast cancer cells, data indicating that specific activation of PR variants increases the expression of cell-cycle-promoting genes, but represses growth inhibitory genes in breast cancer cells, might provide a plausible molecular mechanism underlying the elevated effect of EPT on breast cancer development [34].

### 2.3. Hormonal Contraception (HC) and Breast Cancer

HC is a very common and frequent kind of endocrine intervention and used by approximately 13% of all women between 15 and 49 years worldwide [2]. Studies concerning the effect of oral contraceptives (OC) on breast cancer risk have shown inconsistent results. Outcomes range from no impact on breast cancer risk [35] up to a 50–60% increase in RR associated with ever versus never use of OC [36]. In particular, first start of use at a young age and before bearing the first child seem to result in an increase in breast cancer risk [37].

A large prospective cohort study initiated in 1991 found a substantial increase in breast cancer risk for premenopausal women associated with current or recent use of OC. The relative risks (RR) associated with the use of combined oral contraceptives or progestin-only pills were relatively similar, with RRs of 1.5 (95% CI, 1.0–2.0) and 1.6 (95% CI, 1.0–2.4), respectively [36]. However, the transferability of these results to contemporary HC may be questioned, as the estrogen-doses in combined OCs thirty years ago were considerably higher and new progestins, as well as new application routes of HC, have emerged since then. Today, HC does not only include contraceptive pills, but also intrauterine devices, intradermal implants, patches, injections, and vaginal rings. Regarding the differences in breast cancer risk associated with ET and EPT, the considerable number of new progestin-only contraceptive methods that have emerged over the last few years, as well as combined OCs with new progestins, seem to be of particular interest. As the progestin component seems have an impact on breast cancer risk in HRT, the question arises whether this is also the case in HC.

A large US-American case-control study from 2014 with inclusion of over 1000 breast cancer cases diagnosed between the age of 20 and 49 years compared breast cancer risks associated with former and more contemporary OCs [38]. The overall analysis showed a significantly increased breast cancer risk associated with recent use of OCs within the prior year (OR = 1.5, 95% CI = 1.3–1.9). In particular, for OCs containing high doses of estrogen, ethynodiol diacetate, or triphasic norethindrone, a notably high risk increase was observed, with ORs ranging between 2.6 and 3.1. In contrast, for other types of combined OCs containing low-dose estrogen, no elevation in breast cancer risk was found (OR = 1.0; 95% CI = 0.6–1.7). When associations with tumor characteristics were examined, recent use of OC showed a stronger connection with ERα-positive (OR = 1.7, 95% CI = 1.3–2.1) compared to ERα-negative breast cancers (OR = 1.2, 95% CI = 0.8–1.8). However, the difference was not significant.

More recently, a Danish register study with inclusion of 1.8 million women investigated the impact of contemporary HC methods on breast cancer risk [2]. The study showed an increase in risk depending on the duration of use. While HC use for the duration of one year was associated with a non-significant increase in risk of RR = 1.09 (95% CI; 0.96–1.23), the relative risk after more than ten years of use was elevated to 1.38 (95% CI; 1.26–1.51). As might be suspected, due to the observed effect of progestin in HRT, the extent of the risk increase associated with current or recent use of combined OCs was dependent on the progestin compound, with RRs ranging from 1.0 to 1.6. In particular, RR after use of gestodene products remained significantly elevated, even after adjusting the analysis for the contained estrogen dose of each combined OC product. Current or recent use of progestin-only intrauterine devices was also associated with an increase in breast cancer risk (RR = 1.21; 95% CI; 1.11–1.33).

## 3. Physiological Function of *BRCA1/2* Genes and the Impact of Pathogenic Variants on the Endocrine System

There are two different ways of repairing DNA double strand breaks (DSB): homologous recombination, and nonhomologous end joining [39]. While nonhomologous end joining is more error-prone and predominant in mitotic cells, homologous recombination is the major DNA DSB repair mechanism in meiotic cells [40]. *BRCA1/2* genes belong to a family of DNA DSB repair genes and play an important role in homologous DNA recombination. Hence, several studies have discovered a connection between the function of BRCA genes and ovarian aging [7]. While DNA DSB damage is a common cause of carcinogenesis in rapidly dividing cells, unrepaired damage tends to accumulate in resting or slowly dividing cells like the oocyte of the primordial follicle [7]. Due to the earlier decline of function of the intact allele and the more complex role of BRCA1, ovarian aging is more prominent in carriers of pathogenic variants in *BRCA1* than in BRCA2 [7,39].

The main coordinator of these processes is considered to be ATM, a signal-transducing enzyme with the ability to phosphorylate numerous downstream modulators and thus activate tumor suppressors and DNA repair proteins like *BRCA1* and 2 [7]. ATM-mediated DSB repair has been identified as a cause of ovarian aging, due to the accumulation of DSBs in the primordial follicles as an expression of age-related decrease in DNA DSB repair [7,41]. While both *BRCA1* and 2 genes participate in homologous recombination, the function of *BRCA1* is more complex: in addition to homologous recombination, *BRCA1* is involved in damage sensing and checkpoint activation [39]. This difference has an important effect on the different physiological impacts of pathogenic variants in *BRCA1* and 2 on reproductive aging and the endocrine functions.

The importance of ATM-mediated DNA DSB repair for gonadal functioning is well-known from other genetic diseases including mutations in this pathway, such as Fanconi Anemia (FA). Patients with FA regularly experience premature ovarian insufficiency (POI), and spontaneous pregnancy rates are extremely low [42]. There are several crosslinks between FA and pathogenic variants in *BRCA1/2*: one of the genes responsible for FA, FANCD1, is identical to BRCA2, while other proteins affected in FA interact with *BRCA1* [7,42]. Therefore, it is not surprising that clinical evidence suggests an impact of *BRCA1/2* mutations on ovarian function.

Additionally, there are indications for lower Anti-Müllerian-hormone (AMH) levels and earlier menopause in carriers of BRCA pathogenic variants [43], suggesting a lower ovarian reserve and a higher risk of infertility in this population.

However, clinically significant infertility before the age of 36–37 years due to *BRCA1/2* mutations is supposed to be unlikely [39]. In younger women, the function of the intact allele is still solid. Over time, age-related decline in function of the intact BRCA allele leads to an accumulation of DNA DSBs within the oocytes, which ultimately results in apoptosis and reduction in the ovarian reserve [43]. The loss of function of the intact allele is assumed to begin after the age of 30–35 years [39]. Accordingly, significantly lower AMH-levels in *BRCA1* mutation carriers compared to noncarriers have been observed after the age of 35 years [44]. The age-related decline in function occurs earlier in life for BRCA1, which could explain the more pronounced effect of pathogenic variants in *BRCA1* compared to BRCA2 on ovarian reserve loss [43].

Contrarily, studies have also observed a lower response to ovarian stimulation in BRCA mutation carriers at an earlier age. Oktay et al. reported a lower response to stimulation with letrozole and gonadotropin in *BRCA1* pathogenic variant carriers compared to BRCA mutation-negative and untested women [45]. The protocol was developed in order to reduce estrogen exposure in women with breast cancer who undergo ovarian stimulation for fertility preservation before chemotherapy. In this study, *BRCA1* pathogenic variant carriers had lower response rates compared with controls (OR = 38.3; 95% CI, 4.1–353.4, *p* = 0.001), as a possible expression of occult ovarian insufficiency. Mean ages of BRCA mutation carriers, BRCA negative, and untested women were similar. The age of the 10 included women with pathogenic variants in *BRCA1* ranged between 28 and 37 years, and 8 were less than 36 years old. In the study, women with BRCA2 mutations, however, did not produce a lower number of eggs, which could be explained by the differences in function of *BRCA1* and 2 genes.

## 4. Interaction of *BRCA1/2* and Their Pathogenic Variants with ERα and PR

A comprehensive study published by the Consortium of Investigators of Modifiers of *BRCA1/2* (CIMBA) including 4325 *BRCA1* and 2568 BRCA2 mutation carriers observed a higher proportion of ERα-negative (78%) and PR-negative (79%) cases and a lower frequency of HER2 overexpression (90%) in BRCA1-associated BC compared to sporadic BC [46]. Thus, most BRCA1-related tumors fall within the category of “triple-negative” breast cancer (68%), overlapping with basal-like breast cancers. However, it is important to consider that about 20% of *BRCA1* mutation carriers are positive both for ERα and PR. Thus, *BRCA1* pathogenic variant carriers can be divided into two subgroups, a hormone-dependent and a triple-negative group, and both must be considered specifically. The same study reported that BRCA2-associated BC had a low frequency of ERα-negative (23%), PR-negative (35%), and triple-negative cases (16%), similarly to sporadic breast cancer [46]. Thus, the hormone-dependent subgroup in BRCA2 mutation carriers is even larger (65–77%) than in BRCA1-associated BC. The same study observed that among *BRCA1* mutation carriers, the proportion of ERα-positive and PR-positive tumors increased with age but decreased with age among BRCA2 mutation carriers. However, in every age group, the proportion of ERα-negative tumors was higher in *BRCA1* mutation carriers than non-carriers [46,47]. The second nuclear ER, ERβ, which has been suggested to act as partial ERα antagonist and tumor suppressor both in an ERα-dependent and -independent manner, was reported to be less frequently expressed in BRCA1-associated BC (42%) than in sporadic BC (55%), which might contribute to the elevated BC risk in mutation carriers [48].

With regard to experimental in vitro studies, *BRCA1* gene was shown to inhibit the transcriptional activity of PR in mammary epithelial cells, leading to a diminished activation of PR target genes, whereas knockdown of *BRCA1* increased PR action [49]. A variety of in vitro studies have reported strong molecular interactions between ERα and *BRCA1* [50]. *BRCA1* is able to increase expression of ERα, an interaction which might underlie the high rate of ERα-negativity in *BRCA1* pathogenic variant carriers [51,52]. On the other hand, *BRCA1* was shown to decrease the transcriptional activity of ERα, both by direct binding to this receptor and by inhibition of its acetylation, leading to downregulation of PR expression [53,54]. Furthermore, *BRCA1* gene diminished the activity of AKT, leading to a decreased ligand-independent activation of ERα [55]. Summing up the effects of *BRCA1* on this receptor, even when leading to an increased ERα expression, its strong negative effects on ERα activity suggest that *BRCA1* might act as ERα antagonist. Thus, at the molecular level, the smaller group of ERα-positive *BRCA1* pathogenic variant carriers are suggested to exhibit an enhanced activity of ERα signaling. *BRCA1* and ERα differentially regulate the important proliferation genes C-MYC and CCND1, which are often overexpressed in cancer. ERα is known to induce expression of both genes. However, *BRCA1* does not only suppress ERα activity, but also inhibits expression of C-MYC and CCND1 [56]. Following these experimental data, *BRCA1* mutation carriers with positive ERα status are expected to exhibit increased ERα activity, a positive PR status, and high levels of key proliferation activators.

This is in line with clinical data from comprehensive studies showing an adverse effect of ERα expression on survival of patients with BRCA1-associated BC [57,58]. With regard to tumors of *BRCA2* mutation carriers exhibiting a high frequency of ERα-positivity, both studies also reported expression of this receptor as correlated with shorter survival compared to the ERα-negative BRCA2-related subgroup. The adverse effect of ERα on survival increased with time after diagnosis [59]. Thus, for ERα-positive *BRCA1/2*-related BC, chemoprevention, oophorectomy, and adjuvant treatment with selective estrogen receptor modulators or aromatase inhibitors to reduce risk of contralateral breast cancer are promising options. For chemoprevention, oophorectomy, and adjuvant therapy with drugs blocking estrogen signaling, studies exist that delivered a proof-of-concept [60,61,62]. However, further studies with a longer follow-up are necessary.

On the other hand, the CIMBA study reported a negative ERα status both in *BRCA1* and BRCA2-associated BC as associated with a higher histological grade than positive ERα status tumors (*p* = 1.2 × 10−13 for *BRCA1* and *p* = 0.001 for BRCA2) [46]. In both *BRCA1* and *BRCA2* mutation carriers, grade 3 tumors were less likely to be ERα-positive than grade 1 tumors. A recent meta-analysis reported that particularly the large group of patients with BRCA1-related TNBC had significantly poorer DFS and OS, and regarding BRCA2-associated TNBC, the OS was significantly poorer in studies with >5 years of follow-up [63]. The fact that both positive and negative ERα status exerted adverse effects on patients with *BRCA1/2*-related BC could be explained by the different pathways activating proliferation in ERα-positive and -negative BC cells, the estrogen responsive subgroup mostly using ERα-triggered cyclin activation, and the ERα-negative subgroup proliferating predominantly due to growth factor signaling.

## 5. Impact of Endocrine Interventions on Cancer Risk in *BRCA1/2* Pathogenic Variant Carriers

### 5.1. Risk-Reducing Surgeries and Breast Cancer Risk

*BRCA1/2* pathogenic variant carriers do not only face a high risk of breast, but also of ovarian cancer. Given the poor prognosis and limited options for early detection, BRCA mutation carriers are encouraged to undergo RRBSO after completion of family planning [64]. However, estrogen loss following RRBSO in premenopausal women is associated with several adverse effects that have to be taken into consideration. In the general population, an increase in cardiovascular diseases and all-cause mortality following premenopausal RRBSO has been demonstrated [65]. In addition, a shortening of expected lifespan has been described [66,67]. A recent meta-analysis analyzed data from 20 cohort studies, published between 1998 and 2022, including data from 921,517 women [68]. They found that women entering menopause before the age of 45 had a significantly increased risk of type II diabetes mellitus, hyperlipidemia, coronary heart disease, stroke, and cardiovascular events. However, this negative impact on survival could be counteracted by the use of HRT [65]. Compared to the general population, RRBSO was associated with a decrease in all-cause mortality in BRCA pathogenic variant carriers [69]. Whether additional HRT after RRBSO in BRCA pathogenic variant carriers might be able to decrease mortality even further, by preventing cardiovascular diseases associated with early estrogen loss, has not yet been examined.

When examining the breast cancer risk associated with endocrine interventions in BRCA pathogenic variant carriers, the history of risk-reducing surgeries such as RRBSO and BRRM has to be taken into consideration. In *BRCA1* pathogenic variant carriers, BRRM has proven to decrease breast cancer-specific and all-cause mortality [70]. Even though other studies have also shown a benefit of BRRM for *BRCA2* pathogenic variant carriers, a recent observational study did not confirm an increase in breast-cancer-specific survival for BRRM compared to surveillance in this subgroup [70]. This could be explained by the more favorable tumor characteristics in *BRCA2* pathogenic variant carriers compared to *BRCA1* and therefore the better prognosis in case of early detection.

BRCA1-related breast cancer is more likely to be triple-negative and frequently shows positivity for p53 protein, which is associated with a more aggressive behavior [71]. In contrast, BRCA2-related breast cancer is often ER-positive and, apart from the more frequent contralateral appearance, does not substantially differ from sporadic breast cancer [72]. These differences in tumor characteristics reflect the different functions of *BRCA1* and 2 genes that have been discussed above.

Additional to the risk reduction for ovarian cancer, RRBSO has also been suggested to have an impact on breast cancer risk. A former prospective multi-center cohort study reported a notable decrease in breast cancer risk in both *BRCA1* and 2 pathogenic variant carriers associated with RRBSO, resulting in a decrease in breast-cancer-specific mortality of more than 50% (HR = 0.44; 95% CI, 0.26–0.76) [69]. In contrast, more recent studies questioned the extent of RRBSO-related impact on breast cancer risk, suggesting that the effect of former studies might have been overestimated due to bias and confounding [73]. A large prospective cohort study published in 2020, including 2272 *BRCA1* and 1605 *BRCA2* pathogenic variant carriers, did not observe a protective effect of RRBSO regarding breast cancer in *BRCA1* mutation carriers. In *BRCA2* pathogenic variant carriers, a protective effect was reported depending on the time following RRBSO (HR = 0.51; 95% CI, 0.26–0.99 for ≥5 years after surgery) [73]. These differing results compared to previous studies might be due to various reasons. One explanation could be that the point of time at which RRBSO was performed was too late to attenuate breast cancer risk in this cohort, as the mean age of breast cancer onset is younger for *BRCA1* than for *BRCA2* pathogenic variant carriers. In addition, a reduced ovarian reserve is associated with pathogenic variants in *BRCA1*, as explained above, and thus earlier natural menopause might cause the chosen point of time for RRBSO to be too late for a reduction in breast cancer risk by hormonal deprivation.

### 5.2. HRT and Breast Cancer in BRCA1/2 Pathogenic Variant Carriers

The effect of HRT on breast cancer risk in *BRCA1/2* mutation carriers is of particular importance, given the clear recommendation for HRT after RRBSO in premenopausal women.

According to a recent Italian survey, the majority of *BRCA1/2* pathogenic variant carriers after RRBSO were found to be highly symptomatic regarding menopausal complaints [5]. Nevertheless, more than 65% of the respondents in the survey had never used HRT. Regarding the reasons for why no HRT was taken, the most common answers with 40% each were fear of possibly increased cancer risks and refusal of prescription by the attending physician. At the same time, more than 60% of the respondents did not feel well-informed about the adverse effects of HRT by their healthcare providers. These results clearly show the need for further education regarding endocrine interventions in BRCA pathogenic variant carriers, both in patients and attending doctors.

There are several studies that investigated the safety of HRT in *BRCA1/2* mutation carriers (Table 1). However, the number of included women remained low, especially regarding carriers of pathogenic variants in BRCA2. Concerning risk-reducing surgeries, *BRCA2* mutation carriers were mostly excluded after BRRM. In contrast, HRT in BRRM carriers without RRBSO has hardly been investigated. Survival analyses of pathogenic variant carriers in *BRCA1* or *2* with or without HRT use are not available.

To our knowledge, only one study so far has addressed the safety of HRT in BRCA pathogenic variant carriers without prior RRBSO [74]. Kotsopoulos et al. included 432 case-control pairs, all of which had a *BRCA1* mutation, where 75.7% had undergone natural menopause and BRRM was an exclusion criteria. In this study, no HRT-associated increase in breast cancer risk was observed (OR = 0.80; 95% CI, 0.55–1.16; *p* = 0.24), regardless of whether ET or EPT was used.

A meta-analysis from 2018 included two prospective and one retrospective cohort study with 1100 *BRCA1/2* pathogenic variant carriers after RRBSO [75]. The retrospective cohort study also included a number of women with prior BRRM [76]. However, the total number of mutation carriers in the retrospective cohort study was very low (47 BRCA1, 26 BRCA2).

The meta-analysis did not show an impact of HRT on breast cancer risk, neither in the total cohort (HR = 1.01; 95% CI, 0.16–1.54), nor for the two prospective trials (HR = 0.98; 95% CI, 0.63–1.52). One of the included prospective studies by Kotsopoulos et al. reported a potentially adverse effect for HRT containing progestin [77]. After a follow-up of 10 years, *BRCA1* pathogenic variant carriers who used ET had a significantly lower breast cancer incidence than those who used EPT (12 vs. 22%, *p* = 0.04). In the meta-analysis, this finding could not be confirmed [75]. Nevertheless, in both the entire cohort and in the prospective cohort, there was a non-significant beneficial trend for ET versus EPT regarding breast cancer incidence.

**Table 1:** Comparison of the impact of HRT on breast cancer risk in the general population and *BRCA1/2* pathogenic variant carriers.

Affected Groups	General Population	*BRCA1/2* Pathogenic Variant Carriers
Impact of HRT	Possible increase in risk depending on timing and duration of use Higher increase in risk associated with EPT vs. ET [33]	Lower case numbers, to date no increase in breast cancer risk Most recent meta-analysis: 1100 *BRCA1/2* pathogenic variant carriers after RRBSO [75] Only one study without RRBSO (864 BRCA1, 75.7% natural menopause) [74] Potential beneficial trend for ET vs. EPT

### 5.3. Hormonal Contraception (HC) and Breast Cancer in BRCA Pathogenic Variant Carriers

Several studies have investigated the association between OC and breast cancer risk in *BRCA1/2* mutation carriers, however, with inconsistent results. Some studies found a risk elevation [78], while others did not [79]. The effect of more recent forms of hormonal contraception such as hormonal IUDs in *BRCA1/2* pathogenic variant carriers has not been examined.

A review from 2010 that took into account seven case-controlled and one retrospective cohort study reported a mild to moderate risk increase associated with ever use of OC [78]. Risk was increased further when OC was taken for four or more years before the first full-term pregnancy (HR for *BRCA1* = 1.49; 95% CI, 1.05–2.11. HR for BRCA 2 = 2.58; 95% CI, 1.21–5.49). Similarly, two case-only studies observed an association between breast cancer risk and OC, when OC use was started at an early age or breast cancer was diagnosed below 40 years [80,81].

The most recent original study from 2018 by Schrijver et al. showed similar results [82]. The study consisted of both a prospective and retrospective cohort, the latter divided into a full-cohort and a left-truncated analysis. The prospective cohort included 2276 *BRCA1* pathogenic variant carriers, the retrospective cohorts 5705 (full cohort), and 3828 (left-truncated) *BRCA1* mutation carriers. The numbers for *BRCA2* pathogenic variant carriers were 1610, 3521, and 2512, respectively. For *BRCA1* mutation carriers, an increase in breast cancer risk after ever use of OC was observed in the retrospective analyses, with HRs between 1.26 and 1.39. Furthermore, an inverse correlation with duration of use before the first full-term pregnancy was observed in the left-truncated retrospective analysis for *BRCA1* pathogenic variant carriers below the age of 36 years. However, the findings could not be confirmed in the prospective cohort. For *BRCA2* mutation carriers, an increase in risk after ever use of OC was found, both in the prospective cohort (HR = 1.75; 95% CI, 1.03–2.9) and in the full-cohort retrospective analysis (HR = 1.52; 95% CI, 1.28–1.81). An association with longer duration of use, particularly before the first full-term pregnancy was only observed in the full-cohort retrospective analysis.

Due to discrepancies between their prospective and retrospective analyses, Schrijver et al. considered an actual causal relation to be rather unlikely [82]. However, the differences between the two cohorts might also have been caused by survival bias or an underrepresentation of younger women in the prospective cohort.

A recent meta-analysis, however, found divergent results [83]. The analysis included nine case-controlled studies published between 2002 and 2021. Overall analysis showed a non-significant decrease in breast cancer risk associated with OC (OR = 0.86; 95% CI, 0.70–1.06; *p* = 0.1594). In contrast to earlier publications, this meta-analysis found a risk elevation when OC was first used at the age of 20 years or older (OR = 1.21; 95% CI, 1.07–1.36, *p* = 0.002). However, various limitations complicate a proper interpretation of these data. First of all, the authors themselves suspected that the limited number of suitable studies with different counts of included pathogenic variant carriers and differing observation periods may have lowered the statistical power of the meta-analysis. Furthermore, the wide range of the publication dates indicated a high heterogeneity regarding the used products and associated hormone doses.

A recent modeling study aimed to facilitate decision-making on the use of combined oral contraceptives in carriers of a pV in *BRCA1* or *2* and investigated the risks for breast, ovarian, and endometrial cancer [84]. They found that use of oral contraceptives initially led to an increased risk of breast cancer and in the long term to a reduced risk of ovarian and endometrial cancer [84]. For a hypothetical cohort of 10,000 *BRCA1* mutation carriers, 10 years of continuous combined oral contraceptive use resulted in 99 additional cases of breast cancer by the age of 35 years, in addition to the 572 cases expected for women with no use of oral contraceptives [84].

Overall, even though there have been a number of studies on the subject, data are still limited, especially regarding *BRCA2* mutation carriers. To our knowledge, none of the existing studies evaluated a possible effect of different types and dosages of contraceptive pills in *BRCA1/2* pathogenic variant carriers. This is especially problematic when taking into consideration the considerable amount of new hormonal contraceptive methods on the market and the recent findings concerning the impact of progestin components in HC on breast cancer risk in the general population, as discussed above. For BRCA mutation carriers, hormonal contraception, therefore, involves a high number of uncertainties and should be used after consideration of the individual benefits and risks, in consultation with the person seeking advice (Table 2).

## 6. Conclusions

Endocrine interventions such as hormone replacement therapy (HRT) and hormonal contraception (HC) have become increasingly common over the years. Both interventions are based on the actions of estrogens, particularly on ERα, which is known to promote proliferation of breast cancer cells, and/or progestins, which activate not only the nuclear PR isoforms A and B, known to exert distinct effects on breast cancer growth, but also more recently identified membrane progesterone receptors (mPRs/PAQRs and PGRMCs), which are mediators of highly complex non-genomic progesterone actions with non-fully understood roles in cancer cells [85,86]. Thus, the risks and benefits of such interventions have to be considered carefully by the administering physicians. This is particularly relevant for women who already face a higher cancer risk, especially *BRCA1/2* pathogenic variant carriers.

Large studies have clearly suggested that EPT treatment increased BC risk to a higher extent than treatment with ET alone, a difference which seemed to be even more pronounced for women who started HRT at the age of 60–69 years. With regard to women with pathogenic variants in *BRCA1/2* genes, in contrast, none of the studies examining the effect of HRT on BC and OC risk in this subgroup reported a significant effect of HRT, probably due to the smaller case numbers included. However, reports suggesting an altered expression and activity of ERα and an altered PR A/B ratio in *BRCA1/2* mutation carriers demand not only the clinical determination of these receptor protein subtypes in this BC subgroup, but also necessitate studies including larger *BRCA1/2* case numbers addressing the effect of different HRT regimens on cancer risk in these patients.

The situation with regards to the comparison of the effects of HC on cancer risk in the normal population and on *BRCA1/2* pathogenic variant carriers is similar. Large studies have suggested that the use of oral contraceptives is particularly able to increase BC risk in women with first start of use at a young age and before bearing the first child. The BC risk increase was found to be not only dependent on the duration of use but also elevated for older oral contraceptives containing higher estrogen doses or in the case of combined OCs containing gestodene. In addition to OCs, intrauterine devices with progestins only also led to an increase in breast cancer risk. Again, many initial studies regarding the effect of HC on *BRCA1/2* pathogenic variant carriers led to inconclusive results, due to small case numbers. Later studies and meta-analyses suggested an increase in BC risk, particularly when OC was taken for 4 or more years before the first full-term pregnancy or for breast cancer cases diagnosed below 40 years. However, several limitations of these studies, like the fact that mostly *BRCA1* and to a lesser extent BRCA2 mutation carriers were included, as well as the ongoing development of novel HC therapy regimens, suggest the need for further studies on larger patient collectives using contemporary drugs. In order to improve the quality of the available data, it is imperative that women with pathogenic variants in the *BRCA1* or 2 gene are distinctly included in large registries, such as the HerediCaRe of the German Consortium for Hereditary Breast and Ovarian Cancer, in which all interventions should be recorded in detail, reliably, and prospectively.

In conclusion, to date, no valid assessment regarding potential differences in the effects of different HRT or HC regimens on breast or ovarian cancer risk of *BRCA1/2* pathogenic variant carriers and the general population can be given. A deeper understanding of the observed hormone levels in both groups, and particularly of the roles and ratios of nuclear ER and PR variants and of membrane PRs as targets of HRT and HC progestins, which seem to be altered in women with pathogenic variants in *BRCA1/2*, could lead to an individual risk assessment.

## Figures and Tables

**Table 2:** Comparison of the impact of hormonal contraceptives (HC) on breast cancer risk in the general population and *BRCA1/2* pathogenic variant carriers.

Affected Group	General Population	*BRCA1/2* Pathogenic Variant Carriers
Impact of HC	Increase in risk associated with oral contraceptives, modified by containing progestin Increase in risk associated with progestin-only intrauterine device [2]	Potential increase in risk with oral contraceptives, depending on timing and duration of use Most recent original study: 6030 *BRCA1* and 3809 *BRCA2* [82] No investigations regarding contemporary hormonal contraceptives or different products
